# FOXP1 is associated with oncogenesis and clinical outcomes in hematologic malignancies

**DOI:** 10.3389/fimmu.2025.1569641

**Published:** 2025-07-02

**Authors:** Xiang-mei Wen, Zi-jun Xu, Hao-xi Ni, Su-wan Liu, Ye Jin, Wei Zhao, Shu-yu Luo, Yuan-yuan Fang, Zhen-wei Mao, Jiang Lin, Jun Qian

**Affiliations:** ^1^ Laboratory Center, Affiliated People’s Hospital of Jiangsu University, Zhenjiang, Jiangsu, China; ^2^ Zhenjiang Clinical Research Center of Hematology, Affiliated People’s Hospital of Jiangsu University, Zhenjiang, Jiangsu, China; ^3^ The Key Lab of Precision Diagnosis and Treatment in Hematologic Malignancies of Zhenjiang City, Affiliated People’s Hospital of Jiangsu University, Zhenjiang, Jiangsu, China; ^4^ Department of Hematology, Affiliated People’s Hospital of Jiangsu University, Zhenjiang, Jiangsu, China

**Keywords:** FOXP1, hematological malignancies, prognosis, tumor microenvironment, methylation, immunotherapy

## Abstract

Depending on the cellular context and cancer type, FOXP1 functions as an oncogene or a tumor suppressor. However, the clinical role of FOXP1 in hematologic malignancies has not been studied comprehensively. This study systematically analyzed the association of FOXP1 expression with clinical outcomes, including prognosis and immunotherapeutic response, as well as biological functions across a range of hematological cancers. Our findings demonstrated that FOXP1 expression was dysregulated in several hematological malignancies and was associated with poor prognosis. FOXP1 was highly expressed in acute myeloid leukemia (AML). Methylation of the FOXP1 promoter was significantly reduced in patients with AML compared to the healthy control subjects and those with myelodysplastic syndromes. FOXP1 promoter methylation showed an inverse relationship with FOXP1 gene expression in AML. Moreover, FOXP1 expression was associated with the tumor infiltration of B cells, natural killer cells, and T cells, as well as the cytolytic score across various hematologic malignancies. Our data showed that FOXP1 expression was a promising biomarker for predicting responses to immunotherapy in AML patients. Functionally, the knockdown of FOXP1 demonstrated antileukemic effects, including reduced AML cell proliferation and cell cycle arrest in the G1-S phase. In conclusion, this study systematically investigated the role of FOXP1 across a spectrum of hematological malignancies and demonstrated that FOXP1 was a promising prognostic biomarker and a potential therapeutic target in AML and other hematological malignancies.

## 1 Introduction

Hematological malignancies such as leukemias, lymphomas, histiocyte tumors, and mast cell tumors originate from the blood, lymph nodes, and bone marrow (1, 2). The incidence of hematological cancers has increased significantly because of the aging global population (3). Diffuse large B-cell lymphoma (DLBCL) constitutes approximately 30% of the non-Hodgkin’s lymphoma cases in Western countries and 45.8% of the non-Hodgkin’s lymphoma cases in China, with the 5-year survival rate of DLBCL patients ranging from 32% to 81% (4, 5). The five-year overall survival (OS) rate of patients with acute myeloid leukemia (AML) is less than 50% (6, 7). Recent advances in targeted therapies and immunotherapy have significantly enhanced the survival rates of cancer patients (8). The fundamental principle of targeted therapy and immunotherapy involves the identification of molecular markers that are intricately associated with the disease onset to facilitate the development of targeted therapeutic agents and stratification of patients for personalized treatment with targeted therapeutics (9, 10).

Forkhead box (FOX) proteins are important transcription factors that are characterized by the presence of an evolutionarily conserved winged helix or forkhead box DNA-binding domain of about 90 residues (11–13). In humans, the FOX superfamily includes the FOXP subfamily of transcription factors such as FOXP1, FOXP2, FOXP3, and FOXP4 (14, 15). The *FOXP1* gene is 628 kb in length and is located on chromosome 3p14.1. FOXP1 plays a significant role in several biological processes, including neural development, monocyte differentiation, macrophage function, as well as T- and B-cell development and differentiation (16–18). FOXP1 also plays a key role in heart, lung, and lymphocyte development (16). FOXP1 is downregulated in human glioma (19), hepatocellular carcinoma (HCC) (20), and esophageal squamous cell carcinoma (21). However, FOXP1 is overexpressed in several types of lymphomas such as DLBCL (22, 23), follicular lymphoma (24), primary cutaneous large B-cell lymphomas (25), and gastric mucosa-associated lymphoid tissue lymphoma (26). FOXP1 is a potential biomarker of poor prognosis in patients with DLBCL (27, 28), Furthermore, the FOXP1-GINS1 axis is involved in DLBCL development and doxorubicin resistance (28).

Previous studies have demonstrated that FOXP1 plays contradictory roles in different cancers. It acts as a tumor suppressor in a few cancers, whereas it plays an oncogenic role in other cancers. Therefore, there is a need to perform a molecular pan-blood-cancer analysis of FOXP1 for an in-depth understanding of its role in different blood cancers. In our previous studies, we have conducted comprehensive pan-cancer and pan-hematologic malignancy analyses to explore the clinical, genomic, and immunological features of key immune-related molecules. For instance, we identified the LILRB family as potential immune checkpoints in AML and other malignancies (29). Additionally, we systematically investigated the CD300 family across multiple cancer types, revealing their potential as immune regulators and therapeutic targets in AML (30). More recently, we explored the clinical significance and biological functions of immune-related genes in hematologic malignancies, including STING and TGFB1, highlighting their prognostic value and impact on anti-tumor immunity (31, 32). Building on these previous findings, we aimed to further investigate the role of FOXP1 across hematologic malignancies. Given the complex and context-dependent roles of FOXP1 in different cancers, a systematic analysis of its expression, prognostic significance, and immune-related functions in hematologic malignancies is crucial. Our study integrates bioinformatics analysis with experimental validation to provide novel insights into the role of FOXP1 in AML, DLBCL, and MM, offering potential implications for its clinical application as a biomarker and therapeutic target.

## 2 Materials and methods

### 2.1 Data acquisition

FOXP1 expression levels across cancer cell lines were analyzed using data from the Cancer Cell Line Encyclopedia (CCLE) database (https://www.broadinstitute.org/ccle). We also analyzed FOXP1 expression levels in hematologic malignancies using data from the Hemap dataset (http://hemap.uta.fi/). Comprehensive pan-cancer data for the FOXP1 gene was obtained from the TCGA, TARGET, and GTEx datasets, which were downloaded from the UCSC Xena Browser (https://xenabrowser.net). All the data were downloaded from the Gene Expression Omnibus (GEO) database (https://www.ncbi.nlm.nih.gov/geo/) and curated using custom scripts. Survival data for patients diagnosed with AML, DLBCL, and MM were obtained from various datasets with survival information from the GEO database (https://www.ncbi.nlm.nih.gov/geo/), cBioPortal for Cancer Genomics (http://www.cbioportal.org/), the Genomic Data Commons data portal (https://portal.gdc.cancer.gov/), and the PREdiction of Clinical Outcomes from Genomic Profiles platform (https://precog.stanford.edu/).

### 2.2 Patient samples

The study protocol was approved by the Ethics Committee of the Affiliated People’s Hospital of Jiangsu University (K-20240151-Y). Bone marrow samples were collected from all the participants after the acquisition of written informed consent. Bone marrow mononuclear cells were isolated via density-gradient centrifugation using the Lymphocyte Separation Medium (Solarbio, China). The expression levels of FOXP1 were analyzed in 93 subjects, including 65 *de novo* AML patients and 28 healthy donors. Targeted bisulfite sequencing was used to assess FOXP1 methylation levels in a cohort of 156 individuals, comprising 103 patients with *de novo* AML, 28 patients with MDS, and 25 healthy donors.

### 2.3 RNA isolation, reverse transcription, and RT-qPCR

Total RNA was isolated from the cells using the Trizol reagent (Invitrogen, USA). Subsequently, reverse transcription of the RNA samples was performed to synthesize complementary DNA (cDNA) using the Reverse Transcription Kit (Takara, Japan). Real-time quantitative PCR (RT-qPCR) was then used to quantify the relative expression levels of FOXP1 with the SYBR Green Master Mix (Takara, Japan) in a 7500 Thermal cycler (Applied Biosystems, CA). GAPDH was utilized as the internal reference gene. We used an efficiency-corrected relative quantification method to account for potential differences in primer amplification efficiencies between the target gene (FOXP1) and the reference gene (GAPDH). The specific formula applied was:


$$N_{FOXP1}={\left(E_{FOXP1}\right)}^{\Delta CT\;FOXP1_{\left(contol-experimental\right)}}÷{\left(E_{GAPDH}\right)}^{\Delta CT\;GAPDH_{\left(control-experimental\right)}}$$

This method calculates the fold change in FOXP1 expression in the experimental group relative to the control group, normalized to GAPDH and corrected for the actual amplification efficiencies of both genes. The RT-qPCR primers used for the amplification of the FOXP1 and GAPDH transcripts are listed in **Table 1**.

**Table 1:** Primers and sequences used for RT-qPCR, targeted bisulfite sequencing and RNA interfere.

Primers	Primer sequence (5’to 3’)	Predicted product size (bp)
RT-qPCR primers		
*FOXP1*-F	GGACAGCTCTCAGTCCACAC	272
*FOXP1*-R	AGGTGGGTCATCATGGCTTG	
*GAPDH*-F	GAAGGTGAAGGTCGGAGTC	
*GAPDH*-R	GAAGATGGTGATGGGATTTC	
Targeted bisulfite sequencing primers		
*FOXP1*-BF	GTTGTAGTTATAAAGGGGTAGTTTTTTTTT	226
*FOXP1*-BR	CAACACCCTTAATATTTTTCATATAACAC	
shRNA sequences		
shFOXP1-1	GCAGCAAGTTAGTGGATTAAA	
shFOXP1-2	TGGCTATGATGACACCTCAAGTTAT	
shFOXP1-3	TGCGAAGATTTCCAATCATTT	
shNC	TTCTCCGAACGTGTCACGTAA	

### 2.4 Targeted bisulfite sequencing

Genomic DNA was extracted from the bone marrow cells using the Puregene Blood Core Kit B (Qiagen, Germany). Subsequently, bisulfite modification of the DNA samples was performed using the EpiTect Bisulfite Kit (Qiagen, Germany). The primers based on the bisulfite-converted DNA were designed using the Primer3 software (http://primer3.ut.ee/) and are shown in **Table 1**. Targeted bisulfite sequencing was performed with methods described in our previous study (33).

### 2.5 Bioinformatics analyses

Patients were stratified into high and low FOXP1 expression groups based on the optimal cut-off value determined by the “survminer” package. The “survcomp” package was used to evaluate the prognostic significance of FOXP1 by aggregating *P*-values and hazard ratios (HRs) in the DLBCL, AML, and MM samples. Kaplan–Meier survival curves were used to estimate the OS and event-free survival (EFS) rates between groups using the “survminer” package. The 29 functional gene expression signatures related to various aspects of the TME were obtained from a previous study (34). Single-sample gene set enrichment analysis (ssGSEA) was used to compute the signature scores for the tumor samples in the Hemap database using the “GSVA” R package. The CIBERSORT and MCP algorithms were used to quantify the infiltration of immune cell types across various blood cancers (35). The cytolytic scores and HLA scores for the Hemap dataset were estimated as previously described (36). Subsequently, the ESTIMATE algorithm was used to compute the immune scores, stromal scores, and tumor purity for all the blood cancer types. TIDE algorithm (http://tide.dfci.harvard.edu) was used to determine the potential response to immune checkpoint blockade (ICB). The correlations between the expression levels of FOXP1 and programmed cell death 1/programmed cell death ligand 1 (PD-1/PD-L1) in AML were determined using the BeatAML, TCGA AML, and the GSE6891 datasets. To predict patient responses to ICB therapy in clinical immunotherapy settings, we included three independent immunotherapy cohorts encompassing three cancer types (gastric cancer, metastatic melanoma, and breast cancer). This was done to ascertain whether variations in the efficacy of ICB therapy existed between groups with high and low FOXP1 expression.

### 2.6 Cell culture, cell proliferation, and cell cycle

THP1 cells were cultured in RPMI 1640 medium supplemented with 10% fetal calf serum (ExCell Bio, China) in a humidified incubator maintained at 37°C and 5% CO_2_. Lentivirus-delivered FOXP1 shRNAs were synthesized by HanBio and the specific sequences are listed in **Table 1**. The shRNA transfections were performed using the HitransB-2 Transfection Reagent (Genechem, China) according to the manufacturer’s instructions. Cell proliferation and cell cycle analyses were performed using the Cell Counting Kit-8 (CCK8, Vazyme Biotech, China) and the Cell Cycle Assay Kit (Vazyme Biotech, China), respectively.

### 2.7 Statistical analyses

Statistical analyses were performed using R software. The correlation between two continuous variables was assessed using the non-parametric Spearman test. Categorical variables were compared using the chi-square test or Fisher’s exact test. Data visualization was performed using the “ggplot2” and “survcomp” (for forest plots) R packages. A *P*-value of less than 0.05 was considered statistically significant.

## 3 Results

### 3.1 The expression level of FOXP1 across hematological malignancies

**Figure 1:**
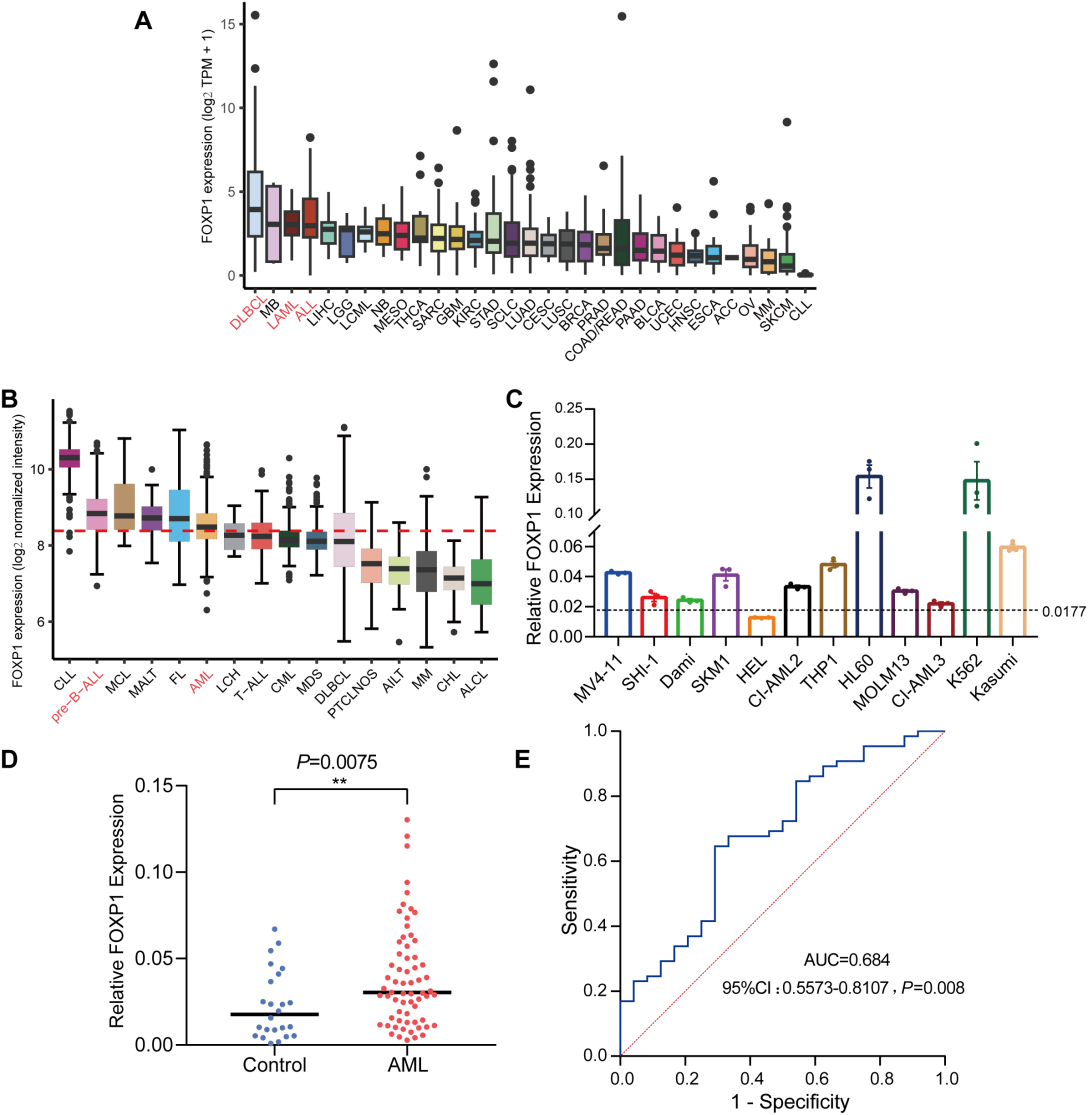
FOXP1 gene expression levels in cell lines and patient samples. **(A)** FOXP1 mRNA expression levels in different tumor cell lines from the CCLE database. **(B)** FOXP1 mRNA expression levels (log_2_ normalized intensity) in samples from patients with different hematological malignancies, based on microarray data from the Hemap database. **(C)** FOXP1 mRNA expression levels in 12 leukemia cell lines, based on RNA-seq data. In both panels, the dotted line indicates the mean FOXP1 expression level across all samples within the respective dataset. **(D)** RT-qPCR analysis of FOXP1 mRNA expression in AML patients (n=65) and normal controls (n=28). **(E)** ROC curve analysis of the diagnostic performance of FOXP1 mRNA expression in AML (*P*=0.008) (**p < 0.01).

Analysis of RNA-seq data from the CCLE database revealed that FOXP1 expression levels (log_2_ TPM + 1) were relatively elevated in hematologic malignancy-derived cell lines, particularly those corresponding to DLBCL, AML, and acute lymphoblastic leukemia (ALL), compared to cell lines from other tumor types (**Figure 1A**). This suggests a potential hematopoietic lineage-specific upregulation of FOXP1. Further analysis using the Hemap database, which contains microarray-based transcriptomic profiles of various hematologic malignancies, showed that FOXP1 expression (log_2_ normalized intensity) was markedly elevated in patient samples of pre-B cell acute lymphoblastic leukemia (pre-B-ALL) and AML (**Figure 1B**). These findings suggest a disease-specific overexpression pattern of FOXP1 in certain hematologic cancers. Given the high expression of FOXP1 across hematological malignancies and the clinical significance of AML, we focused on AML to further investigate FOXP1 expression in cell lines. This allowed us to validate database findings and explore potential functional implications in AML pathogenesis. Next, we assessed the expression levels of FOXP1 in a panel of leukemia cell lines using RT-qPCR and found that FOXP1 expression levels were highest in the HL60, K562, Kasumi, and THP1 cell lines (**Figure 1C**). Furthermore, the relative FOXP1 transcript levels were significantly higher in the AML patients (n=65; range: 0.003 to 0.13) compared to the normal controls (n=28; range: 0.000 to 0.067) (*P*=0.0075; **Figure 1D**). Subsequently, ROC curve analysis showed that the AUC value for distinguishing AML patients from healthy controls based on FOXP1 expression was 0.684 [95% confidence interval (CI), 0.5573–0.8107] (*P*=0.008) (**Figure 1E**). This indicates that FOXP1 expression exhibits differential expression between AML patients and normal controls, although further validation in larger cohorts is required to confirm its diagnostic potential.

### 3.2 Prognostic value of FOXP1 in hematological malignancies

**Figure 2:**
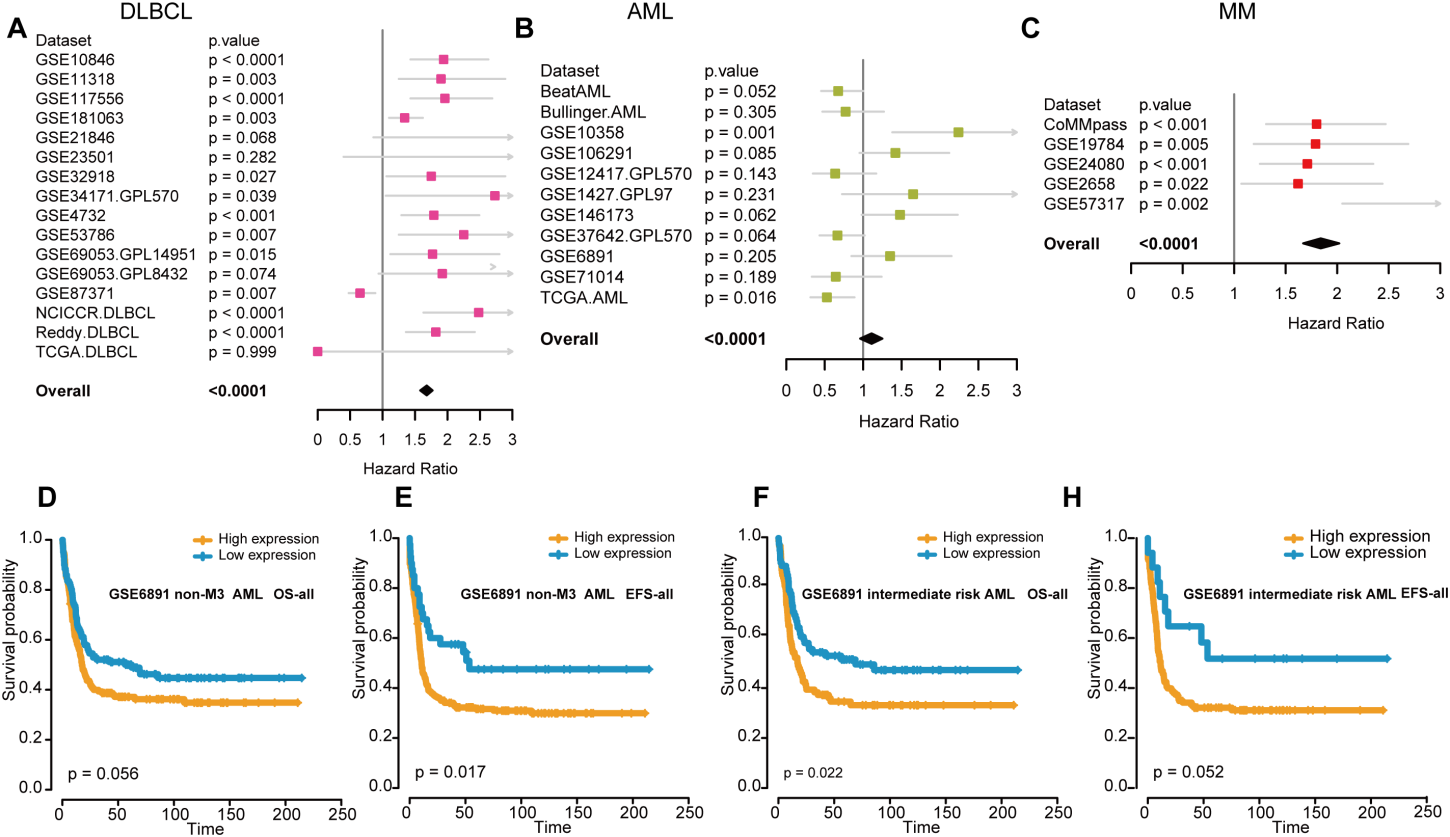
The association between FOXP1 expression and prognosis in hematological malignancies. **(A-C)** Forest plots show the prognostic effects of FOXP1 expression in DLBCL **(A)**, AML **(B)**, and MM **(C)**. The hazard ratios and *P*-values were integrated across datasets, with *P*-values derived from Cox regression analysis in each dataset and combined using the weighted Z-method. **(D, E)** Kaplan-Meier survival curves show the OS and EFS rates for the non-M3 AML patients in the GSE6891 dataset with high or low FOXP1 expression. The high and low groups were defined using the maxstat statistical method. **(F, G)** Kaplan-Meier survival curves show the OS and EFS rates for the intermediate-risk AML patients in the GSE6891 dataset with high or low FOXP1 expression.

Next, we assessed the prognostic value of FOXP1 expression in patients with hematological malignancies. HRs and the corresponding *P* values were estimated using the Cox regression hazard model. FOXP1 expression was associated with a higher risk of DLBCL and MM, but its association with the risk of AML was ambiguous and not statistically significant in the datasets analyzed (**Figures 2A–C**). Therefore, to further elucidate the role of FOXP1 in AML, Kaplan–Meier survival curve analysis was performed using the GSE6891 dataset (**Figures 2D–G**). Kaplan-Meier survival curve results were comparable with the results from the Cox regression analysis of the GSE6891 dataset. Patients in the non-M3 AML group with elevated expression of FOXP1 were associated with significantly lower EFS rates (*P*=0.017). Furthermore, higher expression of FOXP1 was significantly associated with poor OS of patients with intermediate-risk AML (*P*=0.022). These findings suggested that high FOXP1 expression was an indicator of poorer prognosis in AML patients. Therefore, our data suggested that FOXP1 might serve as a potential prognostic biomarker in certain hematologic malignancies, particularly DLBCL and MM. However, its prognostic value in AML remains inconclusive and requires further prospective validation.

### 3.3 The methylation levels of FOXP1 promoter in AML and MDS samples

**Figure 3:**
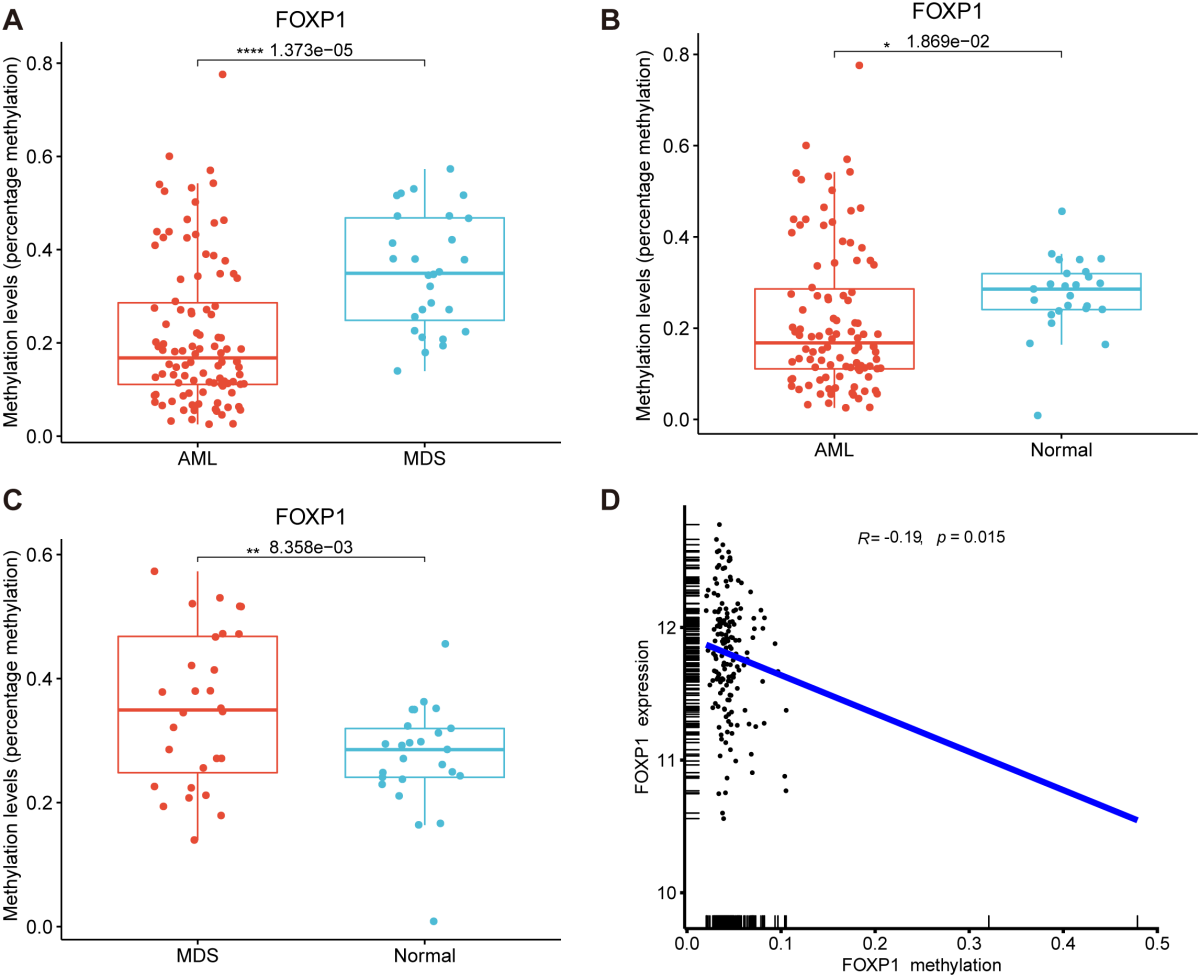
*FOXP1* gene promoter methylation levels in AML and MDS. **(A)** Comparative analysis of *FOXP1* gene promoter methylation levels between AML and MDS samples. **(B)** Comparative analysis of *FOXP1* gene promoter methylation levels between AML and control groups. **(C)** Comparative analysis of *FOXP1* gene promoter methylation levels between MDS and control groups. **(D)** Correlation analysis between *FOXP1* gene promoter methylation and FOXP1 mRNA expression levels in AML (*p < 0.05, **p < 0.01, ****p < 0.0001).

We then performed MethylTarget sequencing of bone marrow samples from 156 human subjects, including 103 patients with AML, 28 patients with MDS, and 25 healthy controls to analyze the status of DNA methylation of 1 CpG sites of *FOXP1* gene promoter (chr3: 71542279). The methylation level of the *FOXP1* gene promoter region was significantly lower in the AML samples compared to samples from patients with MDS and normal controls (*P*<0.001 and *P*<0.05, respectively) (**Figures 3A, B**). Methylation levels in the *FOXP1* gene were significantly higher in samples from patients with MDS compared to the control group (*P*<0.01) (**Figure 3C**). To confirm the relationship between methylation level and gene expression, we analyzed patient data from the TCGA database. We found that FOXP1 expression levels in AML patients were negatively correlated with the FOXP1 promoter methylation levels (R= -0.19, *P*<0.05) (**Figure 3D**). These results suggested that *FOXP1* gene expression was probably regulated by the methylation levels in the *FOXP1* promoter region in AML.

### 3.4 Associations between TME signatures and FOXP1 expression levels across hematological malignancies

**Figure 4:**
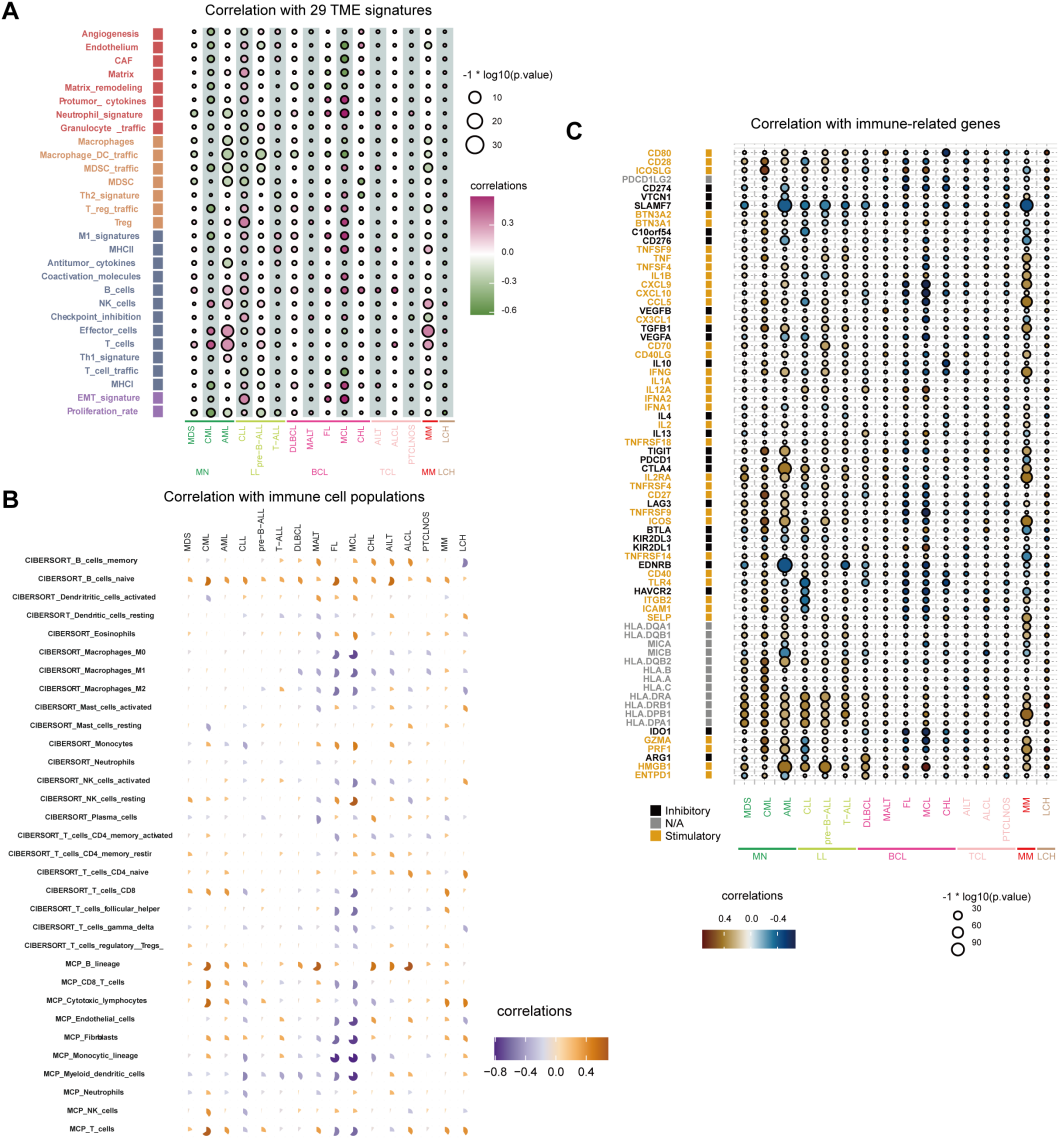
Relationship between immune response indices and FOXP1 expression. **(A)** The bubble chart shows the relationship between FOXP1 expression and 29 TME signatures across various hematological malignancies in the Hemap database. **(B)** CIBERSORT and MCP-counter analysis results show the relationship between FOXP1 expression and immune cell populations in multiple hematologic malignancies. **(C)** The relationship between the expression levels of FOXP1 and various immune-related genes across hematological malignancies.

We analyzed the correlation between FOXP1 expression levels and 29 TME signatures in hematological malignancies. As shown in **Figure 4A**, FOXP1 expression demonstrated a negative correlation with the infiltration of tumor-promoting immune cells, such as macrophages, dendritic cells, and myeloid-derived suppressor cells (MDSCs). Conversely, FOXP1 expression showed a positive correlation with the infiltration of tumor-suppressing immune cells, such as B cells, natural killer (NK) cells, and T cells. Subsequently, we assessed the composition of tumor-associated immune cells using the CIBERSORT and MCP-counter algorithms, both of which produced consistent results. FOXP1 expression showed a positive correlation with the infiltration of antitumor immune cells, including B cells (**Figure 4B**). To further investigate the effect of FOXP1 expression on the tumor immune microenvironment, we analyzed the relationship between the expression levels of FOXP1 and the immune-related genes. In most hematological tumors, FOXP1 expression demonstrated a positive correlation with the expression levels of stimulatory immune genes such as ICOSLG, IL2RA, and HMGB1, and a negative correlation with the expression levels of inhibitory immune genes, such as SLAMF7 (**Figure 4C**).

**Figure 5:**
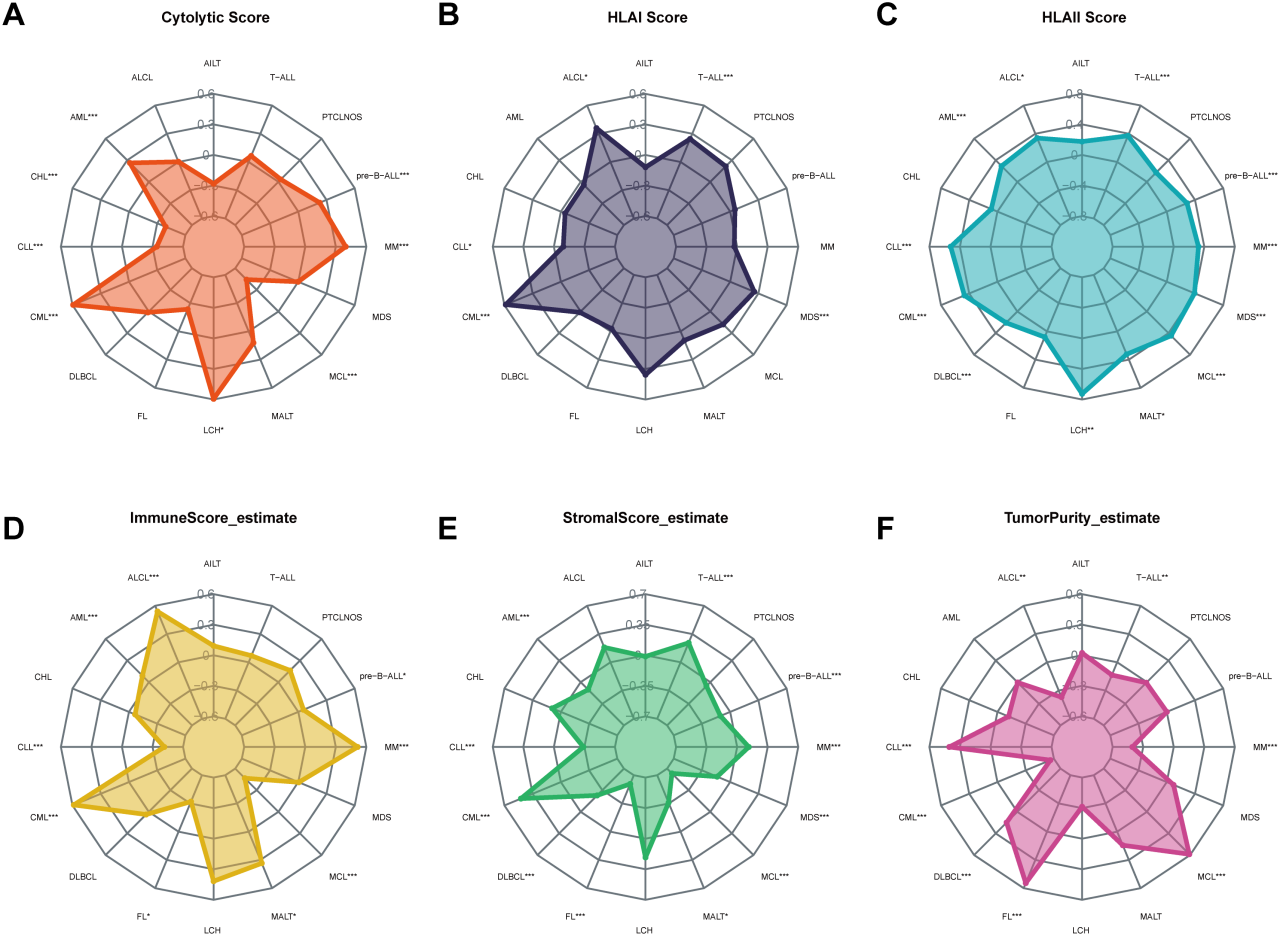
Correlation of FOXP1 expression with the cytolytic score **(A)**, HLA I score **(B)**, HLA II score **(C)**, immune score **(D)**, stromal score **(E)**, and tumor purity **(F)** in hematological neoplasms. Asterisks represent the statistical *P* value (*P < 0.05; **P < 0.01; ***P < 0.001).

The cytolytic score is a useful biomarker for estimating the anti-tumor immune activity. FOXP1 expression showed a positive correlation with the cytolytic score in AML, MM, Langerhans cell histiocytosis (LCH), and chronic myeloid leukemia (CML) (**Figure 5A**). Furthermore, FOXP1 expression showed a positive correlation with the HLA class I and HLA class II scores in CML and MDS (**Figures 5B, C**). FOXP1 expression also showed a positive association with the immune score and a negative correlation with the stromal score in AML. Immune and stromal scores were lowest for MCL, which demonstrated the highest tumor purity (**Figures 5D-F**). These data suggested that FOXP1 expression influenced the growth, development, and progression of blood cancers by modulating the tumor immune microenvironment.

### 3.5 Correlations between FOXP1 expression and immunotherapeutic response

**Figure 6:**
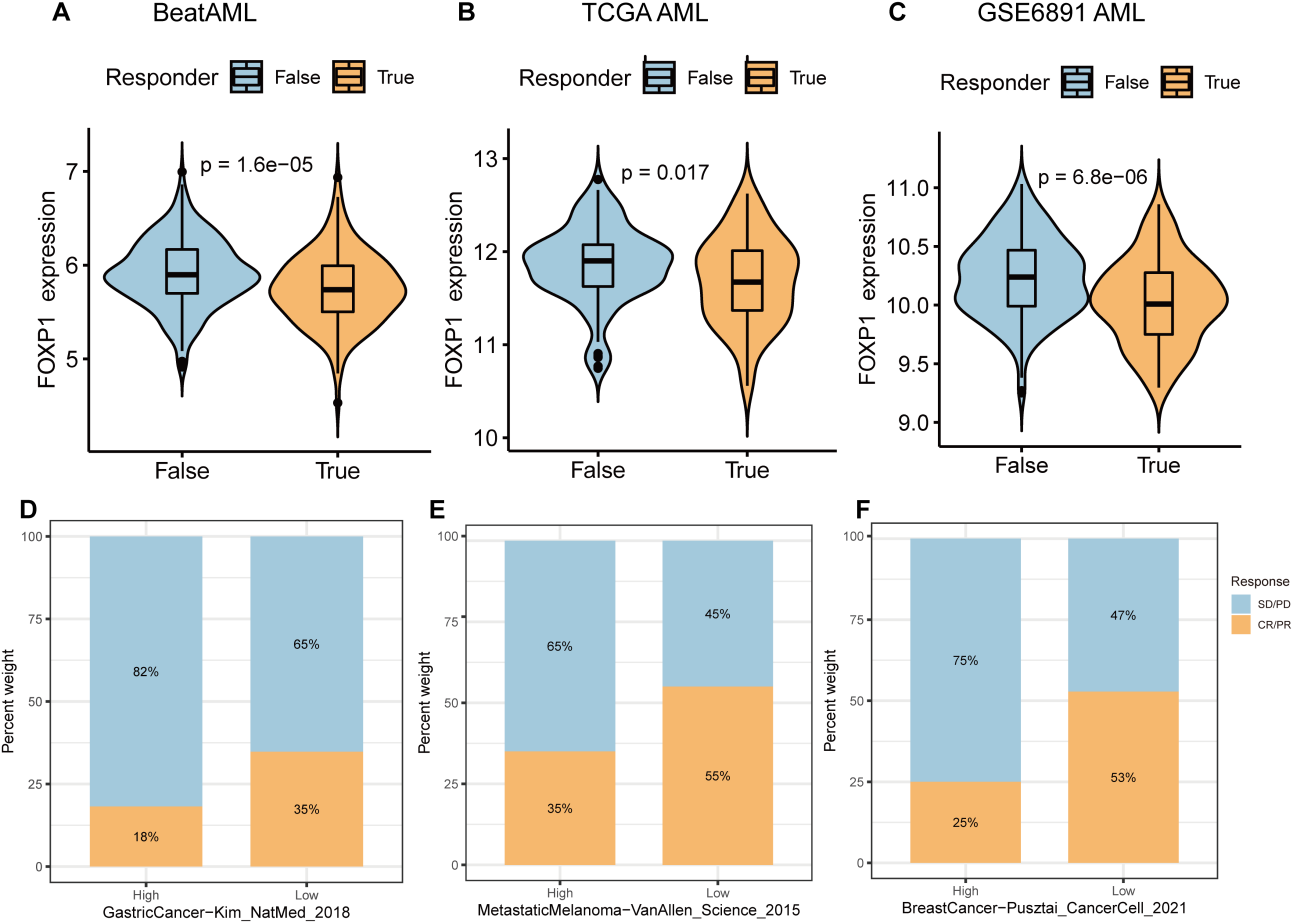
Correlation of FOXP1 expression with immunotherapeutic response in cancer patients. **(A-C)** The expression levels of FOXP1 between immunotherapeutic responders and non-responder groups in the BeatAML **(A)**, TCGA AML **(B)**, and GEO AML (GSE6891) datasets **(C)**. **(D-F)** Bar plots show the percentages of ICB responders and non-responders in the FOXP1-high and FOXP1-low expression groups for the ICB treatment cohorts of gastric cancer, metastatic melanoma, and breast cancer patients. Blue indicates non-responders with stable disease [SD] or progressive disease [PD]; Orange indicates responders with complete response [CR] or partial response [PR].

ICB therapy has revolutionized cancer treatment but is not effective for all patients. Therefore, there is a need to identify effective biomarkers for accurately identifying cancer patients who would benefit from ICB treatment. Towards this, the TIDE algorithm was used to analyze the association between FOXP1 expression and immunotherapy response. FOXP1 gene expression was significantly different between immunotherapy-responsive and immunotherapy-non-responsive groups of patients in the BeatAML (**Figure 6A**), TCGA AML (**Figure 6B**), and GSE6891 datasets (**Figure 6C**). FOXP1 expression was significantly lower in patients responding to immunotherapy compared to the non-responders. TIDE analysis provided a preliminary prediction of immunotherapy response in AML patients; however, the applicability of TIDE in AML remains uncertain. Given the lack of actual immunotherapy response data in AML, we referenced clinical data from solid tumors. We analyzed the expression of FOXP1 in three cohorts of cancer patients who received ICB treatment to determine if FOXP1 expression correlated with their response to therapy. The immunotherapeutic response rate between the high and low FOXP1-expressing gastric cancer patients was not statistically significant (**Figure 6D**). However, in the metastatic melanoma and breast cancer cohorts that underwent immunotherapy, patients with low FOXP1 expression demonstrated a significantly higher therapy response compared to those with high FOXP1 expression (**Figures 6E, F**). Consequently, we propose that FOXP1 is a potential immunotherapeutic response biomarker for some cancer types. Future prospective clinical trials are warranted to validate these findings in AML.

### 3.6 Biological function of FOXP1 *in vitro*

**Figure 7:**
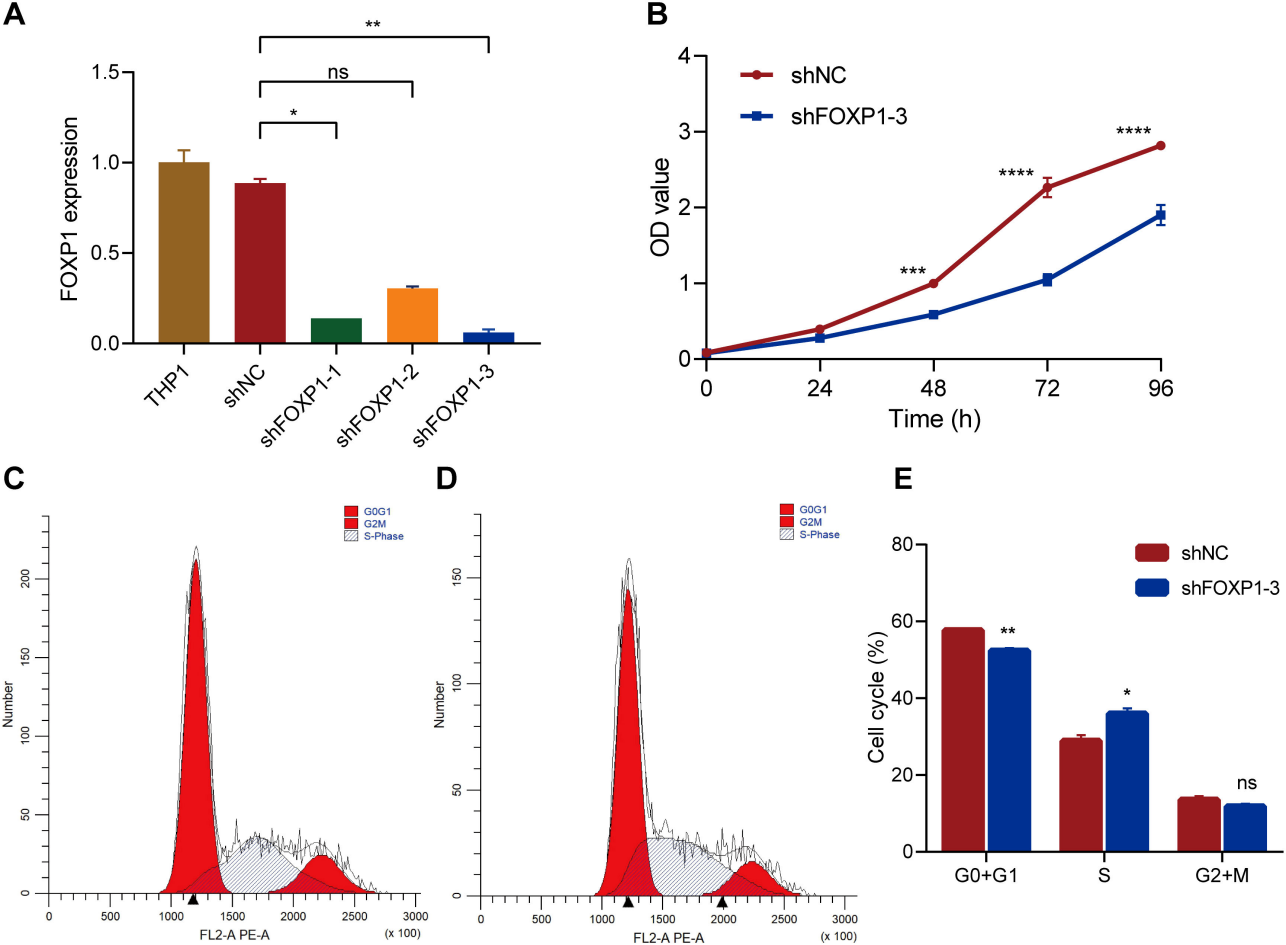
The effects of FOXP1 knockdown on the biological behavior of THP1 cells via lentiviral transduction. **(A)** RT-qPCR results show the relative FOXP1 transcript expression levels in the THP1 cells transfected with shNC, shFOXP1-1, shFOXP1-2, and shFOXP1-3. **(B)** CCK-8 assay results show the proliferation status of the shNC- and shFOXP1-3-transfected THP-1 cells. **(C-D)** Flow cytometry analysis results show the cell cycle phase distribution of THP1 cells transfected with shNC **(C)** and shFOXP1-3 **(D)**. **(E)** The bar graph shows the percentage of G0/G1, S, and G1+M phase cells in the THP-1 cells transfected with shNC and shFOXP1-3 (*p < 0.05, **p < 0.01, ***p < 0.001, ****p < 0.0001, ns, not significant).

THP1 cells were used to perform *in vitro* experiments to analyze the biological functions of FOXP1. THP1 cells were transfected with three FOXP1 short hairpin RNAs (shFOXP1-1, shFOXP1-2, and shFOXP1-3) and the control shRNA (shNC) to generate stable FOXP1-knockdown THP1 cells and the corresponding controls. RT-qPCR analyses were used to confirm the efficacy of the FOXP1 knockdown in the THP1 cell lines. FOXP1 expression levels were significantly lower in the THP1 cell lines transfected with three different FOXP1 shRNAs compared to the shNC-transfected or blank THP1 cells; the most significant downregulation was observed in the shFOXP1–3 group (*P*=0.003) (**Figure 7A**). Therefore, we used the shFOXP1-3-transfected THP1 cells for further experiments. CCK-8 assay was used to evaluate cell proliferation at different time points. CCK-8 assay results demonstrated that the proliferation rate of the shFOXP1-3-transfected THP1 cells was significantly slower than that of the control cells (*P*<0.001) (**Figure 7B**). Thus, FOXP1 knockdown significantly inhibited the proliferation of the THP1 cells. We then performed flow cytometry analysis to assess the differences in cell cycling between the shFOXP1-3- and shNC- transfected THP1 cells. Flow cytometry analysis results demonstrated that FOXP1 knockdown in the THP1 cells decreased the percentage of G0/G1-phase cells (*P*=0.0043) and increased the percentage of S-phase cells (*P*=0.0263) (**Figures 7C-E**). This demonstrated that FOXP1 knockdown caused S-phase cell cycle arrest in the THP1 cells. These data showed that FOXP1 expression affected the proliferation and cell cycling of the leukemia cells.

## 4 Discussion

FOXP1 is a pivotal regulator of numerous neurodevelopmental processes. Mutations or deletions in the *FOXP1* gene result in a neurodevelopmental disorder known as FOXP1 syndrome (37, 38). FOXP1 is also a critical regulator that is required for the normal differentiation and maintenance of mature regulatory T cell (Treg) lineages (39). The impact of FOXP1 on tumor growth, and progression is controversial because it can act as a tumor suppressor or as an oncogene in different types of cancers. For example, FOXP1 acts as a tumor suppressor in breast carcinoma, lung cancer, and prostate cancer (40–42). However, FOXP1 plays a pro-tumorigenic role in DLBCL (28). Furthermore, FOXP1 downregulation inhibits the proliferation of HCC cells by inducing G1/S phase cell cycle arrest (20). Moreover, FOXP1 played a role in the progression of MDS to AML (43). While these findings provide valuable insights into its function in specific malignancies, our study extends beyond individual cancers by systematically analyzing FOXP1 across various hematologic malignancies. This approach allows us to gain a comprehensive understanding of its role at the multi-omics and immune regulation levels. Future studies will be directed toward elucidating the mechanistic pathways underlying FOXP1’s function in different hematologic malignancies, particularly in the context of therapeutic targeting.

FOXP1 promotes the growth of AML cells and is associated with poor prognosis in patients with cytogenetically normal AML (44, 45). This study showed that the expression of FOXP1 was significantly higher in the AML patients and cell lines. Furthermore, we evaluated the prospect of FOXP1 as a prognostic biomarker in blood tumors by analyzing the prognostic information of patients with DLBCL, AML, and MM. Patients with high FOXP1 expression were associated with poor prognosis in DLBCL and MM. Cox regression and Kaplan–Meier analysis showed that high expression of FOXP1 was associated with the poor survival rates of AML patients in the GSE6891 dataset. It is reported that FOXP1 is a negative prognostic indicator in patients with AML who have undergone intensive induction chemotherapy and autologous stem cell transplantation during remission (46). FOXP1 promoted therapeutic resistance in AML cells by enhancing the expression levels of SIRT1, a cellular stress sensor (44). FOXP1 also promoted the development of drug resistance in patients undergoing treatment for ovarian cancer (47).

The field of epigenetics is rapidly evolving, and DNA methylation is one of the most widely researched epigenetic modifications (48, 49). Aberrant DNA methylation is associated with the upregulation of oncogenes and silencing of tumor suppressor genes during tumorigenesis (50). Hu et al. reported that the DNA methylation levels in the *FOXP1* gene did not show statistically significant differences between the NSCLC samples and normal lung tissue samples (51). Luo et al. reported that FOXP1 transcription was inhibited by the binding of circFOXP1 to the FOXP1 promoter and recruitment of DNMT1 (52). To assess the methylation patterns in the *FOXP1* gene among the AML and MDS samples, we used targeted bisulfite sequencing to analyze the DNA methylation status at the CpG sites of FOXP1. The results demonstrated that the DNA methylation level in the *FOXP1* gene was significantly lower among samples from the AML patients compared to the samples from the MDS patients and the healthy controls. Furthermore, FOXP1 methylation levels correlated negatively with the FOXP1 mRNA expression values. This suggested that DNA methylation regulated FOXP1 gene expression. However, further investigations are necessary to understand the in-depth mechanism through which promoter methylation regulates FOXP1 gene expression.

Numerous studies have demonstrated that the FOXP family plays a crucial role in modulating the tumor immune environment by activating or inhibiting specific immune molecules (53). FOXP1 suppresses immune responses and downregulates the expression of MHC class II molecules in DLBCL (27). Our data demonstrated a positive correlation between FOXP1 expression and the infiltration of antitumor immune cells, including B cells. B cell-mediated anti-tumor activity primarily depended on the secretion of antibodies targeting the tumor-associated antigens (TAAs) and facilitated activation of the TAA-specific CD4+ T cells through the co-stimulatory signals (54). The cytolytic activity score is an effective predictor of antitumor immunity and prognosis of cancer patients (55). Our data showed that the cytolytic score correlated positively with FOXP1 expression in AML, CML, and MM. Our findings suggested that FOXP1 expression modulated the tumor immune microenvironment by regulating the infiltration of the immune cells in AML, CML, and MM. However, further functional studies are necessary to validate this hypothesis.

We also investigated the potential of FOXP1 expression as a predictive biomarker for the immunotherapy response. Predicted responders exhibited significantly lower FOXP1 expression compared to the non-responders in AML. Furthermore, analysis of ICB therapy datasets suggested that cancer patients with low FOXP1 expression demonstrated higher ICB response rates and improved survival outcomes, including OS and progression-free survival in comparison to cancer patients with high FOXP1 expression. Therefore, patients exhibiting low FOXP1 expression are promising candidates for ICB treatment. A key limitation of our study is that our findings on the association between FOXP1 expression and immunotherapy response are primarily based on bioinformatics analyses of publicly available datasets. While these results suggest that FOXP1 may serve as a predictive biomarker, additional clinical validation and functional studies are required to confirm its role in immunotherapy response. Due to time constraints, we were unable to perform further experimental verification in this study. In future research, we plan to incorporate patient-derived samples and *in vitro*/ex vivo models to elucidate the mechanistic basis of FOXP1’s involvement in immunotherapy response and validate its clinical relevance.

To investigate the biological role of FOXP1 in the hematologic tumor cells, we performed loss-of-function experiments in the THP1 cells. The knockdown of FOXP1 suppressed the proliferation of the THP1 cells. This indicated that FOXP1 served as an oncogene in the THP1 cells. Naudin et al. also reported that the inhibition of FOXP1 suppressed the proliferation of AML cells (45). Furthermore, our study showed that FOXP1 knockdown induced cell cycle arrest at the S phase. Wang et al. demonstrated that the downregulation of FOXP1 inhibited the proliferation of HCC cells by inducing G1/S phase cell cycle arrest (20). FOXP1 also facilitated the proliferation of pre–B acute lymphoid leukemia cells and enhanced their resistance to chemotherapeutic agents (56). These data demonstrated that FOXP1 functioned as an oncogene in AML and promoted tumor cell growth and progression by modulating cell cycle progression. A potential limitation of our study is that the functional role of FOXP1 was primarily investigated in THP1 cells, despite the initial verification of FOXP1 expression in multiple AML cell lines. Although THP1 cells were selected due to their high FOXP1 expression, we acknowledge that validating our findings in additional AML cell lines would further strengthen our conclusions. Due to time constraints, we were unable to conduct these additional experiments in the current study. However, future research will focus on expanding functional analyses to other AML cell lines to provide more comprehensive insights into the role of FOXP1 in AML pathogenesis.

Although our study demonstrates the potential role of FOXP1 as a diagnostic and prognostic biomarker and a therapeutic target through bioinformatics analysis and *in vitro* experiments, we acknowledge the lack of direct clinical and *in vivo* experimental validation. Prospective clinical studies and animal models are crucial to further confirm the diagnostic and therapeutic value of FOXP1 in AML and other cancers. Due to time constraints, we were unable to include these experiments in the current study. In future research, we aim to conduct longitudinal clinical studies to assess FOXP1 expression in patient cohorts and evaluate its prognostic significance. Additionally, we plan to employ animal models to investigate the functional role of FOXP1 in leukemogenesis and its potential as a therapeutic target.

## 5 Conclusions

FOXP1 was highly expressed in AML and correlated with a worse prognosis. FOXP1 promoter methylation regulated FOXP1 gene expression in AML. FOXP1 expression was associated with distinct TME characteristics across various blood cancer types. Also FOXP1 expression was a promising biomarker for predicting responses to immunotherapy. *In vitro*, functional studies demonstrated tumor-promoting effects of FOXP1 in AML. Further research is needed to elucidate the functional role of FOXP1 in hematologic malignancies.

## Data Availability

The raw data supporting the conclusions of this article will be made available by the authors, without undue reservation.
